# Two new species of the genus *Ptychoptera* Meigen, 1803 (Diptera, Ptychopteridae) from Yunnan, China with remarks on the distribution of Chinese species

**DOI:** 10.3897/zookeys.1070.58859

**Published:** 2021-11-11

**Authors:** Xiao Zhang, Zehui Kang

**Affiliations:** 1 Key Lab of Integrated Crop Pest Management of Shandong Province, College of Plant Health and Medicine, Qingdao Agricultural University, Qingdao 266109, China Qingdao Agricultural University Qingdao China

**Keywords:** Chinese fauna, new taxa, phantom crane flies, Ptychopterinae, taxonomy

## Abstract

Sixteen known species of the genus *Ptychoptera* Meigen, 1803 have been recorded from China, of which three occur in Yunnan Province. Herein, two new species from Yunnan, *P.cordata***sp. nov.** and *P.yunnanica***sp. nov.**, are described from China. An updated key to *Ptychoptera* is presented for all Chinese species.

## Introduction

The genus *Ptychoptera* Meigen, 1803 is the largest of the three extant genera in the family Ptychopteridae, with nearly 80 known species (Kang et al. 2019; Boardman 2020). Sixteen *Ptychoptera* species have been previously recorded from China, of which ten species were published recently by Kang et al. (2013) and Kang et al. (2019). The genus *Ptychoptera* can be diagnosed from the other two genera by the following characters: larvae metapneustic and eucephalous, body segments with serially arranged hairs arising from small papillae, and terminal of abdomen with a long retractable respiratory siphon; adults with 13–14 flagellomeres, wing mostly with infuscation, 3 branches of medial vein reaching wing margin, and gonopod with a simple gonocoxite and a varied gonostylus (Rozkošný 1997; Nakamura and Saigusa 2009; Fasbender 2014).

Adults of *Ptychoptera* are often found in sediments rich in organic matter at the margins of streams and reservoirs, or wet forests on the mountains (Wolf et al. 1997; Wolf and Zwick 2001). The adults usually have a long emergence from early spring to late autumn. For example, adults of *P.circinans* Kang, Xue & Zhang, 2019 were collected from March to August, *P.bannaensis* Kang, Yao & Yang, 2013 from April to June and *P.yankovskiana* Alexander, 1945 from June to September (Kang et al. 2013; Kang et al. 2019).

Yunnan Province is situated at the junction of the eastern Asia monsoon region, the tropical monsoon region of southern Asia, the Indo-China and the Tibetan Plateau region (Yang et al. 2004). As one of 25 global biodiversity conservation hotspots, Yunnan is rich in biotic resources (Myers et al. 2000; Myers 2003). Three species of *Ptychoptera* from Yunnan, *P.bannaensis*, *P.lushuiensis* Kang, Yao & Yang, 2013 and *P.wangae* Kang, Yao & Yang, 2013, were first found and described by Kang et al. (2013). Since then, further new materials of the genus from Yunnan became available. In this paper, two new species from Yunnan are described and illustrated. An updated key to *Ptychoptera* species from China is also presented.

## Materials and methods

Specimens were collected by net or by Malaise trap and kept in 75% alcohol. Photographs were taken by a Canon EOS-77D and EF 100 mm f/2.8L IS USM. Genitalia were prepared by boiling the apical portion of the abdomen in lactic acid for 0.5–1.0 hours; then they were examined, and illustrations prepared, using a ZEISS Stemi 2000-C stereomicroscope. After examination, the removed abdomen was transferred to fresh glycerine and stored in a microvial pinned to the respective specimen. Type specimens are deposited in the Entomological Museum of Qingdao Agricultural University, Qingdao, China (QAU) and the Entomological Museum of China Agricultural University, Beijing, China (CAU). Morphological terminology is based primarily on McAlpine (1981) and Fasbender (2014).

## Taxonomy

### Key to *Ptychoptera* from China

**Table:** 

1	Wing with r-m arise from R_4+5_, Rs not longer than r-m (Figs 1c, 3c)	**2**
–	Wing with r-m arise from Rs before or at fork, Rs at least 1.5 times length of r-m	**9**
2	Mesopleuron mostly brown; epandrial clasper brown	***P.circinans* Kang, Xue &** Z**hang, 2019 (Fujian)**
–	Mesopleuron uniformly yellow; epandrial clasper uniformly yellow	**3**
3	Gonostylus long and slender, about 1.5 times length of gonocoxite	***P.bannaensis* Kang, Yao &** Y**ang, 2013 (Yunnan)**
–	Gonostylus short, as long as gonocoxite (Figs 2c, 4c)	**4**
4	Postnotum dark brown with a big yellow spot (Figs 1b, 3b)	**5**
–	Postnotum uniformly black	**6**
5	Wing with spots at forks of R_1+2_, R_4+5_ and M_1+2_ forming a band (Fig. 1c); abdomen with first tergum yellow with caudal 1/5 light brown (Fig. 1a); subapical spine of epandrium absent (Fig. 2b); anterior lobe of basal lobe of gonostylus not bilobate, medial lobe of basal lobe of gonostylus not bilobate (Fig. 2c); apical process of paramere semilunar, apex expanding outward (Fig. 2c)	***P.cordata* sp. nov**. **(Yunnan)**
–	Wing with spots at forks of R_1+2_, R_4+5_ and M_1+2_ separated (Fig. 3c); abdomen with first tergum dark brown with basal 1/5 yellow (Fig. 3a); subapical spine of epandrium transverse conical (Fig. 4b); anterior lobe of basal lobe of gonostylus bilobate, medial lobe of basal lobe of gonostylus bilobate (Fig. 4c); apical process of paramere hook-shaped, apex incurvated (Fig. 4c)	***P.yunnanica* sp. nov. (Yunnan)**
6	Wing with a distinct spot at fork of R_4+5_, spots at forks of R_1+2_ and M_1+2_ weak and nearly invisible	***P.lii* Kang, Yao &** Y**ang, 2013 (Guizhou)**
–	Wing with three distinct spots at forks of R_1+2_, R_4+5_ and M_1+2_ separated or forming a band	**7**
7	Second tergum anterior margin yellow with a median brown spot; medial lobe of basal lobe of gonostylus slender, finger-shaped	***P.lushuiensis* Kang, Yao &** Y**ang, 2013 (Yunnan)**
–	Second tergum anterior margin yellow brown; medial lobe of basal lobe of gonostylus board, tongue-shaped	**8**
8	Abdomen with 5^th^ and 6^th^ terga dark brown, 6^th^ and 7^th^ sterna mostly brown; apical stylus of gonostylus hook-shaped	***P.emeica* Kang, Xue &** Z**hang, 2019 (Sichuan)**
–	Abdomen with 5^th^ and 6^th^ terga mostly yellow, 6^th^ and 7^th^ sterna yellow; apical stylus of gonostylus finger-shaped (Nakamura and Saigusa 2009)	***P.formosensis* Alexander, 1924 (Taiwan; Japan)**
9	Mesopleuron yellow	**10**
–	Mesopleuron black	**12**
10	Wing with bands and clouds	**11**
–	Wing without band or cloud	***P.wangae* Kang, Yao &** Y**ang, 2013 (Yunnan)**
11	Base of Rs with an elliptic cloud; abdomen with sterna yellow	***P.qinggouensis* Kang, Yao &** Y**ang, 2013 (Neimenggu)**
–	Base of Rs without cloud; abdomen with sterna black	***P.clitellaria* Alexander, 1935 (Sichuan)**
12	Epandrial lobes merged with epandrial claspers	**13**
–	Epandrial lobes not merged with epandrial claspers	**17**
13	Wing with r-m its own length before fork of Rs; epandrial claspers short and blunt	***P.separata* Kang, Xue &** Z**hang, 2019 (Xizang)**
–	Wing with r-m close to fork of Rs; epandrial claspers slender	**14**
14	Wing with an elliptic cloud at middle of CuA_1_	**15**
–	Wing without an elliptic cloud at middle of CuA_1_	**16**
15	Epandrial claspers curved downward, tip bifurcated	***P.gutianshana* Yang &** C**hen, 1995 (Zhejiang)**
–	Epandrial claspers straight, tip not bifurcated	***P.bellula* Alexander, 1937 (Jiangxi)**
16	Gonostylus much longer than gonocoxite	***P.xinglongshana* Yang, 1996 (Gansu)**
–	Gonostylus not longer than gonocoxite	***P.longwangshana* Yang &** *C* **hen, 1998 (Zhejiang)**
17	Abdomen with 2^nd^ and 3^rd^ terga brownish black; epandrial claspers finger-shaped and broad basally, curved inwards at middle	***P.lucida* Kang, Xue &** Z**hang, 2019 (Xinjiang)**
–	Abdomen with 2^nd^ and 3^rd^ terga mostly yellow; epandrial claspers flat and acinaciform, middle of inner edge slightly swollen (Kang et al. 2019)	***P.yankovskiana* Alexander, 1945 (Neimenggu; Korea)**

#### Ptychoptera cordata sp. nov.

Taxon classificationAnimaliaDipteraPtychopteridae

FE6184F2-25AF-5A83-BAA1-48045EA04B1C

http://zoobank.org/6568C6B6-5683-4597-9544-7EEECE70DE88

Figs 1 , 2

##### Material examined.

China•1♂, ***holotype*** Yunnan Province, Menghai District, Mengbang Reservoir; 21°54'50"N, 100°17'36"E; 1272 m; 6 Jun. 2019; Z. Kang leg.; QAU•1♂, ***paratype*** same collection data as for preceding; QAU.

##### Diagnosis.

Postnotum dark brown, mediotergite with a cordiform yellow spot; wing marked with two brown bands; apical process of paramere semilunar, apex expanding outward; lateral extension of terminal division of hypandrium triangular with a pair of semicircular lobes medially, terminal division of hypandrium umbelliform.

##### Description.

**Male**. Body length 7.5–8.0 mm, wing length 7.0–7.5 mm.

Vertex and frons brown; face and clypeus yellow with light brown hairs; gena yellow with a black elliptical spot medially, hairs on gena brown; occiput yellow. Compound eyes black without pubescence. Scape, pedicel and basal 2/3 of 1^st^ flagellomere yellow, remaining flagellomeres brown, setae dark brown. Proboscis light yellow with light brown hairs. Palpus yellow with last segment gradually darked apically, hairs brown.

**Figure 1.**
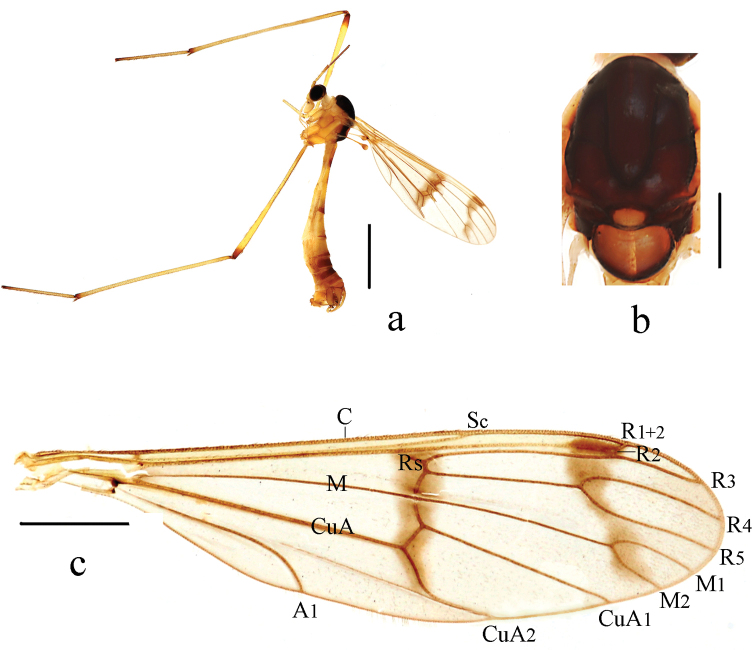
*Ptychopteracordata* sp. nov. **a** habitus of male, lateral view **b** thorax, dorsal view, **c** wing. Scale bars: 2.0 mm (**a**); 0.5 mm (**b**); 1.0 mm (**c**).

Thorax (Fig. 1a, b). Pronotum and propleuron light yellow. Prescutum, scutum and paratergite uniformly black. Scutellum mostly dark brown, middle area yellow with a patch of dense brown hairs. Mediotergite of postnotum dark brown, middle area with a cordiform yellow spot. Laterotergite half brown, outer half yellow with dense dark brown hairs, in dorsal view. Mesopleuron and metapleuron uniformly yellow. Coxae and trochanters yellow; femora yellow with brown ring apically; tibiae yellow with brown ring apically; 1^st^ tarsomere of fore and hind legs yellow brown with brown ring apically. Hairs on legs brown. Wing (Fig. 1c) 3.7 times as long as wide, subhyaline, marked with two brown bands as follows: median band extending from basa of Rs to middle section of CuA_2_; subapical band extending from tip of R_1_, covering pterostigma and extending to tip of M_2_. Veins brown; Sc ending in C at level of basal 1/3 of R_2+3_; Rs straight, slightly shorter than r-m; r-m arise from R_4+5_. Halter and prehaltere pale yellow with brown hairs.

Abdomen (Fig. 1a). First tergum yellow with caudal 1/5 light brown, 2^nd^ tergum yellow with middle area and caudal 1/6 brown, 3^rd^ and 4^th^ terga yellow with posterior margin brown, 5^th^ tergum yellow with caudal 1/2 brown, 6^th^ and 7^th^ terga brown with posterior margin yellow. Sterna yellow. Hairs on abdomen brown.

**Figure 2.**
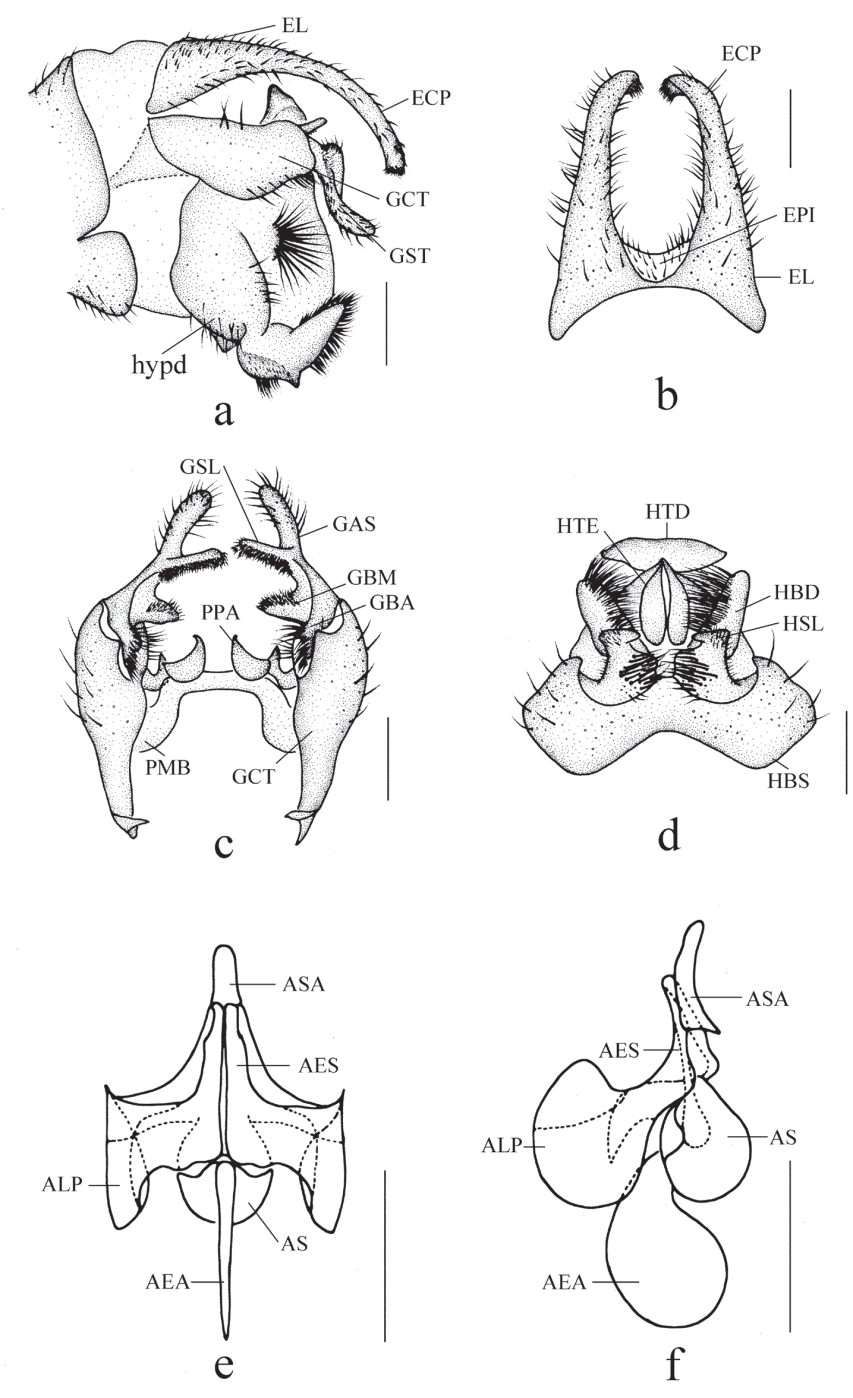
*Ptychopteracordata* sp. nov. **a** male genitalia, lateral view **b** epandrium, dorsal view **c** gonocoxite and gonostylus, dorsal view **d** hypandrium, ventral view **e** aedeagus, anterior view **f** aedeagus, lateral view. Scale bars = 0.2 mm. (AEA = Ejaculatory Apodeme, AES = Aedeagal Sclerite, ALP = Lateral Ejaculatory Process, AS = Sperm Sac, ASA = Subapical Sclerite of Aedeagus, ECP = Epandrial Clasper, EL = Epandrial Lobe, EPI = Epiproct, GAS = Apical stylus of gonostylus, GBA = Anterior lobe of basal lobe of gonostylus, GBM = Medial lobe of basal lobe of gonostylus, GCT = Gonocoxite, GSL = Secondary lobe of Apical Stylus of Gonostylus, GST = Gonostylus, HBD = Basal Division of Hypandrium, HBS = Basal scale of Hypandrium, HSL = Spathate Lobe of Hypandrium, HTD = Terminal Division of Hypandrium, HTE = Lateral Extension of Terminal Division, hypd = hypandrium, PMB = Paramere Base, PPA = Apical Process of Paramere).

Male genitalia (Fig. 2) yellow. Epandrium (Fig. 2b) bilobed, epandrial lobe broad, epandrial clasper tapering and curved ventrally to the middle, slender apically, with brown hairs; epiproct with short hairs. Gonocoxite (Fig. 2c) long and stout, 3.3 times as long as wide, basal apodeme small; apical process of paramere semilunar, apex expanding outward. Gonostylus (Fig. 2c): anterior lobe of basal lobe of gonostylus on inner side with dense long hairs; medial lobe of basal lobe of gonostylus tongue-shaped with dense short hairs on posterior margin; secondary lobe of apical stylus of gonostylus finger-shaped with dense short hairs; apical stylus of gonostylus finger-shaped with short hairs. Hypandrium (Fig. 2d): basal scale of hypandrium dumbbell-shaped basally with several hairs; spathate lobe of hypandrium broad at base with dense long hairs on inner side, hook-shaped apically with short hairs on posterior margin; basal division of hypandrium finger-shaped with dense long hairs on inner side; lateral extension of terminal division of hypandrium triangular with a pair of semicircular lobes medially; terminal division of hypandrium umbelliform. Aedeagus (Fig. 2e, f): subapical sclerite tongue-shaped, apex of subapical sclerite round; aedeagal sclerites with apex laterally compressed, with dorsal corner extended anterodorsally, straight sided and convergent, base broad; lateral ejaculatory processes with base straight, narrow, extended straight anterolaterally; discoid apodemes with elongate ovoid dorsal extension; sperm sac subspherical; ejaculatory apodeme flag-like, closely associated with aedeagal sclerites, larger than sperm sac, paralleling anterior margin of sperm sac.

**Female.** Unknown.

##### Distribution.

China (Yunnan).

##### Etymology.

Specific name from Latin, *cordata* (adjective, feminine, meaning “cordate”), referring to the postnotum with a cordiform spot.

##### Remarks.

This new species is similar to *P.formosensis* from China and Japan but can be separated from the latter by the postnotum dark brown with a cordiform yellow spot, the apical stylus of the gonostylus finger-shaped, and the apical process of paramere semilunar with apex expanding outward. In *P.formosensis*, the postnotum is uniformly black, the apical stylus of the gonostylus is flat tongue-like, and the apical process of the paramere is bilobed (Nakamura and Saigusa 2009). This new species is also similar to *P.annandalei* Brunetti, 1918 from Burma, but can be separated from the latter by the apical process of paramere semilunar with apex expanding outward, the apical stylus of the gonostylus and the secondary lobe of the apical stylus of the gonostylus finger-shaped, not crossing. In *P.annandalei*, the apical lobes of the paramere are dorsoventrally flattened and semicircular with the spine curved anteriorly, apices of the apical stylus and secondary lobe of the gonostylus are crossing (Fasbender 2014). This new species is also similar to *P.perbona* Alexander, 1946 from Burma, but can be separated from the latter by the mediotergite with a cordiform yellow spot, the apical stylus of the gonostylus finger-shaped, the apex of the terminal division of the hypandrium with an umbelliform lobe. In *P.perbona*, the mediotergite is uniformly black, the apical stylus of the gonostylus is stylate, the apex of the terminal division of the hypandrium has a pair of needle-like lateral lobes (Alexander 1946; Fasbender 2014).

#### Ptychoptera yunnanica sp. nov.

Taxon classificationAnimaliaDipteraPtychopteridae

A1A65B4A-59D4-5EFA-A487-A9DC372B6F94

http://zoobank.org/A8B3BF32-C722-423F-9FBB-641D0A78B1BB

Figs 3 , 4

##### Material examined.

China•1♂, ***holotype*** of *P.yunnanica*; Yunnan Province, Binchuan District, Mt. Jizu; 25°56'38"N, 100°23'58"E; 1875 m; 1 Jun. 2019; Z. Kang leg.; QAU•1♀, ***paratype*** of *P.yunnanica*; same collection data as for preceding; QAU•1♀, ***paratype*** of *P.yunnanica*; Yunnan Province, Panlong District, Yunnan Agricultural University; 25°8'4"N, 102°45'2"E; 1953 m; 28 Apr.–3 Jun. 2016.; L. Wang leg.; Malaise trap; CAU•1♀, ***paratype*** of *P.yunnanica*; Yunnan Province, Panlong District, Yunnan Agricultural University; 25°8'9"N, 102°45'8"E; 1958 m; 28 Apr.–3 Jun. 2016; L. Wang leg.; Malaise trap; CAU•1♂, ***paratype*** of *P.yunnanica*; Yunnan Province, Panlong District, Yunnan Agricultural University; 25°8'22"N, 102°45'14"E; 1965 m; 28 Sep.–24 Nov. 2016; L. Wang leg.; Malaise trap; CAU.

##### Diagnosis.

Postnotum dark brown, mediotergite with a big yellow spot; wing marked with three weak brown clouds at tip of R_1_, fork of R_4+5_ and fork of M_1+2_ and a brown band extending from basal of R_2+3_ to middle section of CuA; subapical spine of epandrium transverse conical; basal scale of hypandrium rectangular, anterior margin strongly concaved medially, basal division of hypandrium finger-shaped with dense long hairs basally and medially; terminal division of hypandrium cordiform.

##### Description.

**Male**. Body length 6.5–8.0 mm, wing length 7–7.5 mm.

Vertex and frons dark brown with brown hairs; face and clypeus yellow with light brown hairs; gena yellow with a black elliptical spot medially, hairs dark brown; occiput yellow. Compound eyes black without pubescence. Scape, pedicel and basal 1/2 of 1^st^ flagellomere yellow, remaining flagellomeres brown, setae on antenna dark brown. Proboscis light yellow with brown hairs. Palpus yellow with terminal of last segment brown, hairs brown.

**Figure 3.**
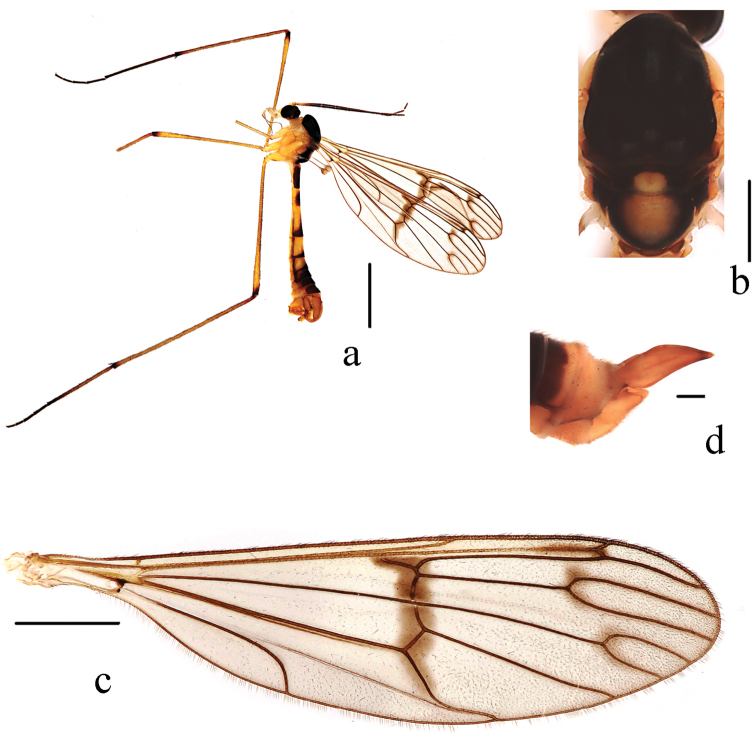
*Ptychopterayunnanica* sp. nov. **a** habitus of male, lateral view **b** thorax, dorsal view **c** wing **d** female terminalia, lateral view. Scale bars: 2,0 mm (**a**); 0,5 mm (**b**); 1,0 mm (**c**); 0,2 mm (**d**).

Thorax (Fig. 3a, b). Pronotum and propleuron light yellow. Prescutum, scutum and paratergite uniformly black. Scutellum mostly brown, middle area yellow with a patch of dense dark brown hairs. Mediotergite of postnotum dark brown, middle area with a big yellow spot. Upper half of laterotergite dark brown, lower half of laterotergite yellow, with brown hairs, in dorsal view. Mesopleuron and metapleuron uniformly yellow. Coxae and trochanters yellow; fore femur yellow and gradually darkened apically; mid and hind femora yellow with brown ring apically; tibiae yellowish brown with brown ring apically; 1^st^ tarsomere of fore and mid legs brown, 1^st^ tarsomere of hind leg yellow with a narrow dark brown ring apically, other tarsomeres dark brown. Hairs on legs dark brown. Relative length of 1^st^ to 5^th^ tarsomeres in hind leg as 7: 2: 1.2: 1: 1.

Wing (Fig. 3c) 3.8 times as long as wide, subhyaline, marked with three brown clouds and a brown band as follows: three weak brown clouds at tip of R_1_, fork of R_4+5_ and fork of M_1+2_; median band extending from basa of Rs to middle section of CuA_2_. Veins brown; Sc ending in C not at level of basal 1/3 of R_2+3_; Rs straight, slightly longer than r-m; r-m arise from R_4+5_. Halter and prehaltere pale yellow with brown hairs.

Abdomen (Fig. 3a). First tergum dark brown with basal 1/5 yellow, 2^nd^ tergum yellow with middle area and caudal 1/6 dark brown, 3^rd^ tergum yellow with caudal 1/5 dark brown, 4^th^ tergum yellow with caudal 1/4 dark brown, 5^th^ to 7^th^ terga dark brown. Sterna yellow. Hairs on abdomen brown.

**Figure 4.**
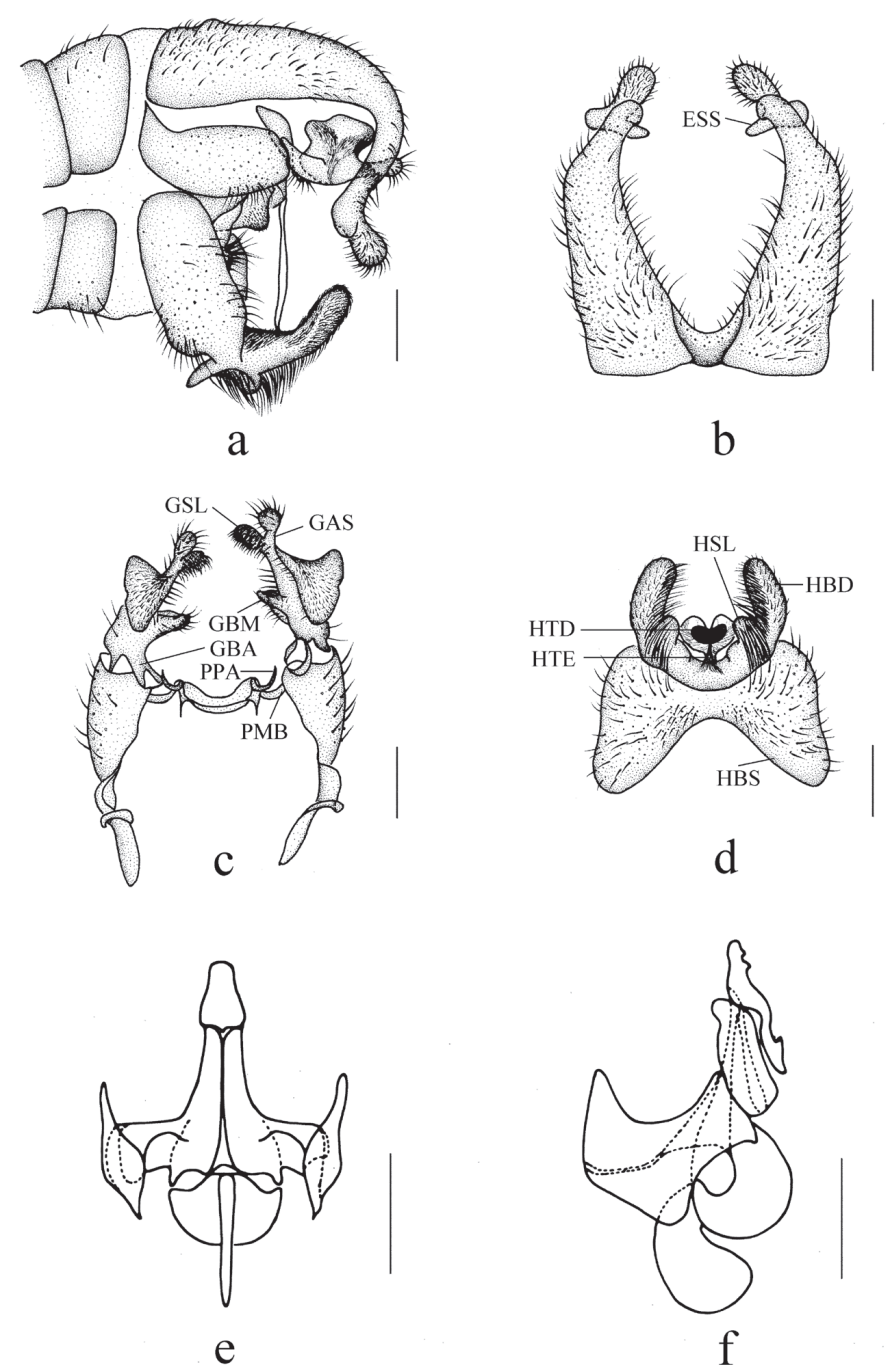
*Ptychopterayunnanica* sp. nov. **a** male genitalia, lateral view **b** epandrium, dorsal view **c** gonocoxite and gonostylus, dorsal view **d** hypandrium, ventral view **e** aedeagus, anterior view **f** aedeagus, lateral view. Scale bars: 0.2 mm. (ESS = Subapical Spine of Epandrium, GAS = Apical stylus of gonostylus, GBA = Anterior lobe of basal lobe of gonostylus, GBM = Medial lobe of basal lobe of gonostylus, GSL = Secondary lobe of Apical Stylus of Gonostylus, HBS = Basal scale of Hypandrium, HSL = Spathate Lobe of Hypandrium, HTD = Terminal Division of Hypandrium, HTE = Lateral Extension of Terminal Division, hypd = hypandrium, PMB = Paramere Base, PPA = Apical Process of Paramere).

Male genitalia (Fig. 4) yellow. Epandrium (Fig. 4b) bilobed, epandrial lobe broad, epandrial clasper tapering distally and curved downward, slightly swollen apically; subapical spine of epandrium transverse conical, with brown hairs; epiproct with short hairs. Gonocoxite (Fig. 4c) short and slender, 2 times as long as wide, basal apodeme 3/4 as long as gonocoxite; apical process of paramere hook-shaped, apex incurvated. Gonostylus (Fig. 4c): anterior lobe of basal lobe of gonostylus bilobate without hairs; medial lobe of basal lobe of gonostylus finger-shaped on middle of inner side with short hairs; secondary lobe of apical stylus of gonostylus fan-shaped basally without hairs, finger-shaped apically with dense short setae; apical stylus of gonostylus finger-shaped with dense short hairs. Hypandrium (Fig. 4d): basal scale of hypandrium rectangular, anterior margin strongly concaved medially; spathate lobe of hypandrium papillary without hairs; basal division of hypandrium finger-shaped with dense long hairs basally and medially; lateral extension of terminal division thin; terminal division of hypandrium cordiform. Aedeagus (Fig. 4e, f): subapical sclerite tongue-shaped, slightly caved bilaterally, apex of subapical sclerite round; aedeagal sclerites with apex laterally compressed, with dorsal corner extended anterodorsally, straight sided and convergent, base broad; lateral ejaculatory processes with base straight, narrow, extended straight anterolaterally; apodemes with elongate quadrangular dorsal extension; sperm sac subspherical; ejaculatory apodeme flag-like, closely associated with aedeagal sclerites, subequal to sperm sac, paralleling anterior margin of sperm sac.

**Female.** Body length 8.5–9.0 mm, wing length 9.0–9.5 mm. Similar to male. Third tergum yellow with caudal 1/3 brown, 4^th^ and 5^th^ terga brown, 6^th^ tergum brown with caudal 1/3 yellow, 7^th^ and 8^th^ terga yellow. Terminalia (Fig. 3d): 8^th^ sternum yellow, 2.8 times as long as 7^th^ sternum; cercus yellow with brown end, blade-shaped, 10^th^ tergum + cercus 1.8 times as long as 8^th^ sternum.

##### Distribution.

China (Yunnan).

##### Etymology.

Specific name *yunnanica* (adjective, feminine) referring to the type locality, Yunnan.

##### Remarks.

This new species is similar to *P.persimilis* Alexander, 1947 from Burma, but can be separated from it by have a mediotergite with a big yellow spot, the subapical spine of the epandrium transverse conical, the apical process of the paramere hook-shaped with the apex incurvated. In *P.persimilis*, the mediotergite does not have a big yellow spot, only the adjoining portion of the scutellum is yellow; the epandrium is nematoform, the apex is expanded and slipper-like, the paramere has elongate, ribbon-like spines directed posterior, crossing medially (Alexander 1947; Fasbender 2014). This new species is also similar to *P.praescutellaris* Alexander, 1946 from Burma, but can be separated from it by having the subapical spine of the epandrium transverse conical, and the secondary lobe of the apical stylus finger-shaped. In *P.praescutellaris*, the apex of the epandrial clasper is bulbous and without a conical projection, and the secondary lobe of the apical stylus is subtrapezoidal (Alexander 1946; Fasbender 2014). This new species resembles somewhat *P.emeica* from China but can be easily separated from the latter by the postnotum dark brown with a big yellow spot, the basal 1/3 of the yellow second abdomen tergum with a median brown spot, the subapical spine of the epandrium transverse conical, and the medial lobe of the basal lobe of the gonostylus finger-shaped. In *P.emeica*, the postnotum is uniformly black, the basal 1/3 of the second tergum of the abdomen is uniformly brown, the epandrium has no transverse conical subapical spine, and the medial lobe of the basal lobe of the gonostylus is broad and tongue-shaped (Kang et al. 2019). Finally, the new species can be separated from *P.lushuiensis* from China by the postnotum dark brown with a big yellow spot, the subapical spine of the epandrium transverse conical, the apical process of the paramere hook-shaped with the apex incurvated, and the terminal division of the hypandrium cordiform. In *P.lushuiensis*, the postnotum is uniformly black, the surstylus of the epandrium does not have a transverse conical subapical spine, the apical process of paramere is sclerotized triangular, and the terminal division of hypandrium cordiform is X-shaped (Kang et al. 2013).

### Distribution of *Ptychoptera* species in China

**Figure 5.**
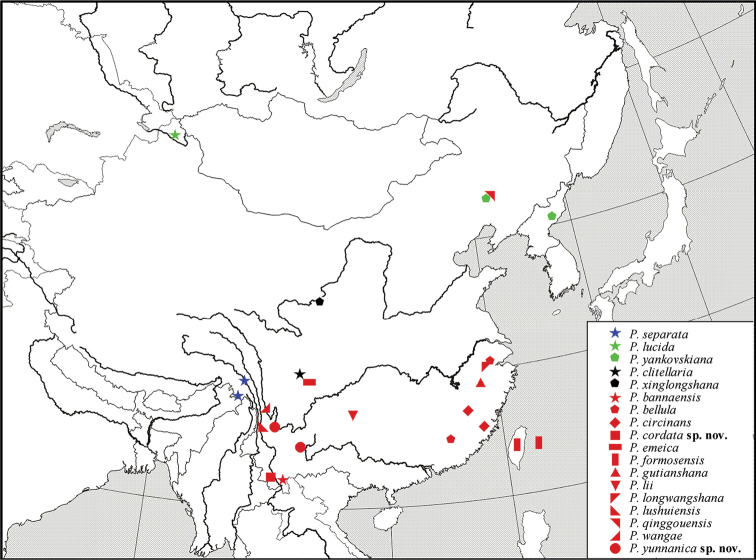
Distribution map of *Ptychoptera* from China.

In total, 18 species of *Ptychoptera* are recorded from China. According to Fasbender (2014), the *Ptychoptera* fauna of China is a complex of the *P.tibialis* group, the *P.contaminata* group, the Southeast Asian *Ptychoptera* and unplaced species. One species, *P.separata*, is endemic to Tibet, China and belongs to the *P.tibialis* group, which otherwise is mainly distributed in the Indian Subcontinent. Two species, *P.lucida* and *P.yankovskiana*, can clearly be characterized as species of the *P.contaminata* group and are distributed in the East Palaearctic region. Twelve Southeast Asian *Ptychoptera* species, namely *P.bannaensis*, *P.bellula*, *P.circinans*, *P.cordata*, *P.emeica*, *P.formosensis*, *P.gutianshana*, *P.lii*, *P.longwangshana*, *P.lushuiensis*, *P.wangae*, *P.yunnanica* are widely distributed in Southwest and East China. *Ptychopteraqinggouensis* has the same characteristics as species of Southeast Asian *Ptychoptera* and is distributed in Inner Mongolia. Two species with insufficient material or illustrations for placement, *P.clitellaria* and *P.xinglongshana*, are distributed in Gansu and Sichuan, respectively (Fig. 5).

## Supplementary Material

XML Treatment for Ptychoptera cordata

XML Treatment for Ptychoptera yunnanica
